# Recurring radiation-induced angiosarcoma of the breast that was treated with paclitaxel chemotherapy: a case report

**DOI:** 10.1186/s40792-020-0790-7

**Published:** 2020-01-16

**Authors:** Yoko Suzuki, Kohei Taniguchi, Minami Hatono, Yukiko Kajiwara, Yuko Abe, Kengo Kawada, Takahiro Tsukioki, Mariko Kochi, Keiko Nishiyama, Takayuki Iwamoto, Hirokuni Ikeda, Tadahiko Shien, Naruto Taira, Masahiro Tabata, Hiroyuki Yanai, Hiroyoshi Doihara

**Affiliations:** 1 Department of Breast and Endocrine surgery in Okayama University Japan Hospital, 2-5-1 Shikatacho Kitaku, Okayama-shi, Okayama-ken 700-8558 Japan; 2 Department of Pathological diagnosis, Okayama University Japan Hospital, 2-5-1 Shikatacho Kitaku, Okayama-shi, Okayama-ken 700-8558 Japan; 3 Department of Hematology, Oncology, Respiratory, and Allergy Medicine, Okayama University Japan Hospital, 2-5-1 Shikatacho Kitaku, Okayama-shi, Okayama-ken 700-8558 Japan

**Keywords:** Radiation-induced angiosarcoma, Radiotherapy, Breast-conserving surgery, Breast cancer, Paclitaxel therapy, Adjuvant therapy of angiosarcoma

## Abstract

**Background:**

Angiosarcoma of the breast is very rare and can be divided into primary and secondary angiosarcoma. Radiation-induced angiosarcoma (RIAS) is classified as secondary angiosarcoma. Diagnosis of RIAS is difficult due to its rarity, and the interpretation of pathological imaging is complicated. In the National Comprehensive Care Network (NCCN) guidelines, the first choice of treatment is surgery with negative margins. Adjuvant radiotherapy (RT) for close soft tissue margins should be considered. Preoperative or adjuvant chemotherapy of nonmetastatic disease is not recommended for angiosarcoma. We report a case of RIAS, which was impossible to diagnose with core needle biopsy (CNB) but was diagnosed by excisional biopsy. The patient was then administered adjuvant chemotherapy using conjugated paclitaxel (PTX).

**Case presentation:**

A 62-year-old woman noticed a tumor in her right breast. She had a history of right breast cancer and had undergone breast-conserving surgery, RT, and tamoxifen therapy 8 years previously. CNB, which was performed twice, was inconclusive. The tumor was surgically excised and pathological analysis yielded a diagnosis of angiosarcoma. She then underwent a right mastectomy. One month after she underwent right mastectomy, a nodule reappeared on the skin of her right breast, and excisional biopsy revealed recurrence of angiosarcoma. A few weeks later another nodule reappeared near the post-operative scar and excisional biopsy revealed recurrence of angiosarcoma. We assumed that surgical therapy was insufficient because the patient experienced relapse of angiosarcoma after complete mastectomy. After the second recurrence, we treated her with systemic chemotherapy using PTX. There was no evidence of recurrence 8 months after chemotherapy.

**Conclusion:**

Although angiosarcoma is difficult to diagnose, many patients have a poor prognosis. Therefore, prompt treatment intervention is desired. Moreover, there is little evidence regarding adjuvant therapy of angiosarcoma since it is a rare disease. We consider that adjuvant therapy helped to effectively prevent recurrence in the patient after complete excision.

## Background

Angiosarcoma is an extremely rare, malignant, blood vessel tumor. Angiosarcoma of the breast can be divided into primary angiosarcoma and secondary angiosarcoma. Radiation-induced angiosarcoma (RIAS) is classified as secondary angiosarcoma. In patients undergoing breast-conserving surgery (BCS) with adjuvant radiotherapy, the estimated incidence of RIAS is 0.05–0.3% [1]. In the last few decades, BCS followed by radiotherapy (RT) has become one of the standard treatments. In addition, postoperative radiotherapy has been performed in many cases with positive axillary node metastasis. Moreover, the number of patients who receive RT is estimated to increase in the future [2]. However, due to difficulties in the diagnosis of angiosarcoma with fine needle aspiration (FNA) or core needle biopsy (CNB), misdiagnosis is frequently reported, delaying the radical surgery [3, 4]. Nonmetastatic angiosarcoma is treated with surgical resection [5]. However, evidence regarding the use of adjuvant therapy for the treatment of breast angiosarcoma is limited and needs careful consideration. Here, we describe a case of angiosarcoma in a patient, who underwent a mastectomy for primary breast cancer 8 years prior and was treated for recurring angiosarcoma with chemotherapy using paclitaxel (PTX).

## Case presentation

**Fig. 1:**
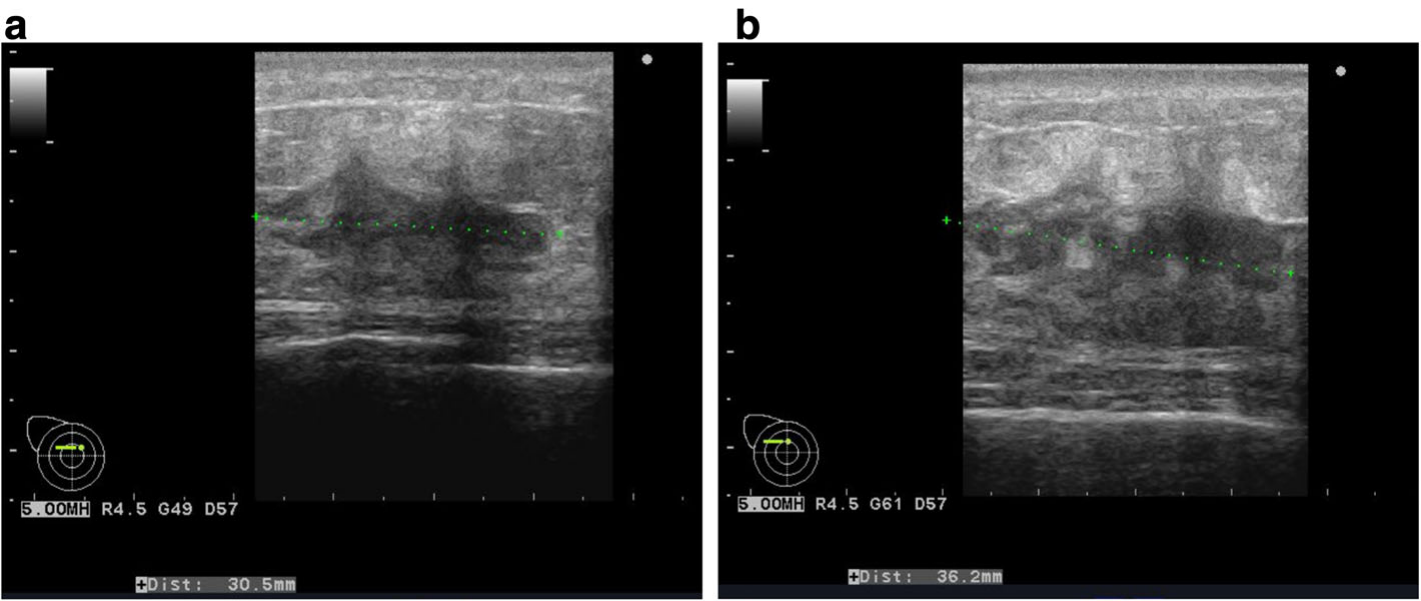
**a** Ultrasonographic images of the patient with the tumor. The inside of the tumor had a low echoic area, and the tumor borders were unclear. **b** The tumor grew larger 2 months later

**Fig. 2:**
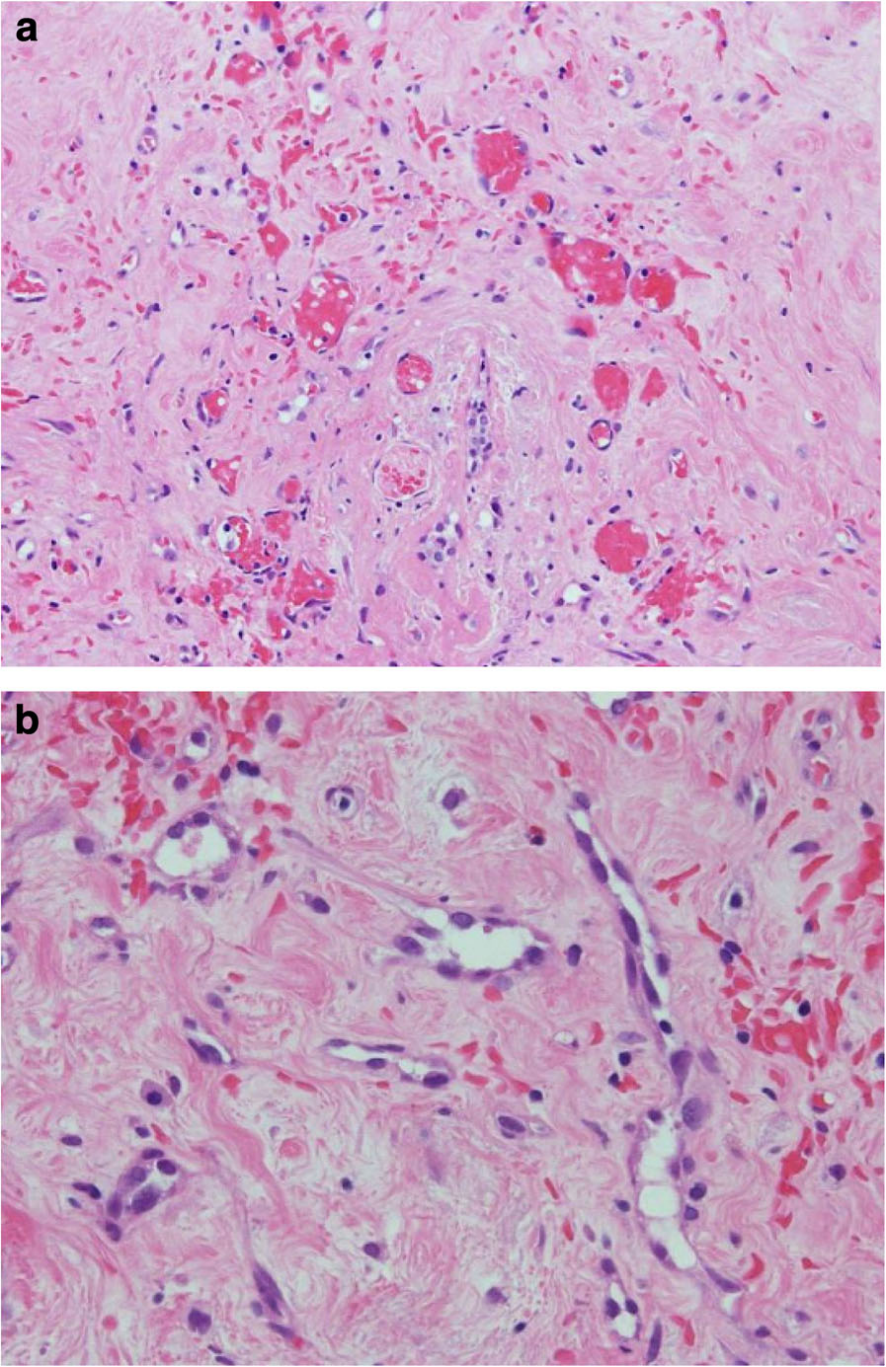
Pathological analyses of core-needle-biopsy. The endothelial cells were swollen; however, atypical epithelial cells were absent. **a** × 40; **b** × 200

**Fig. 3:**
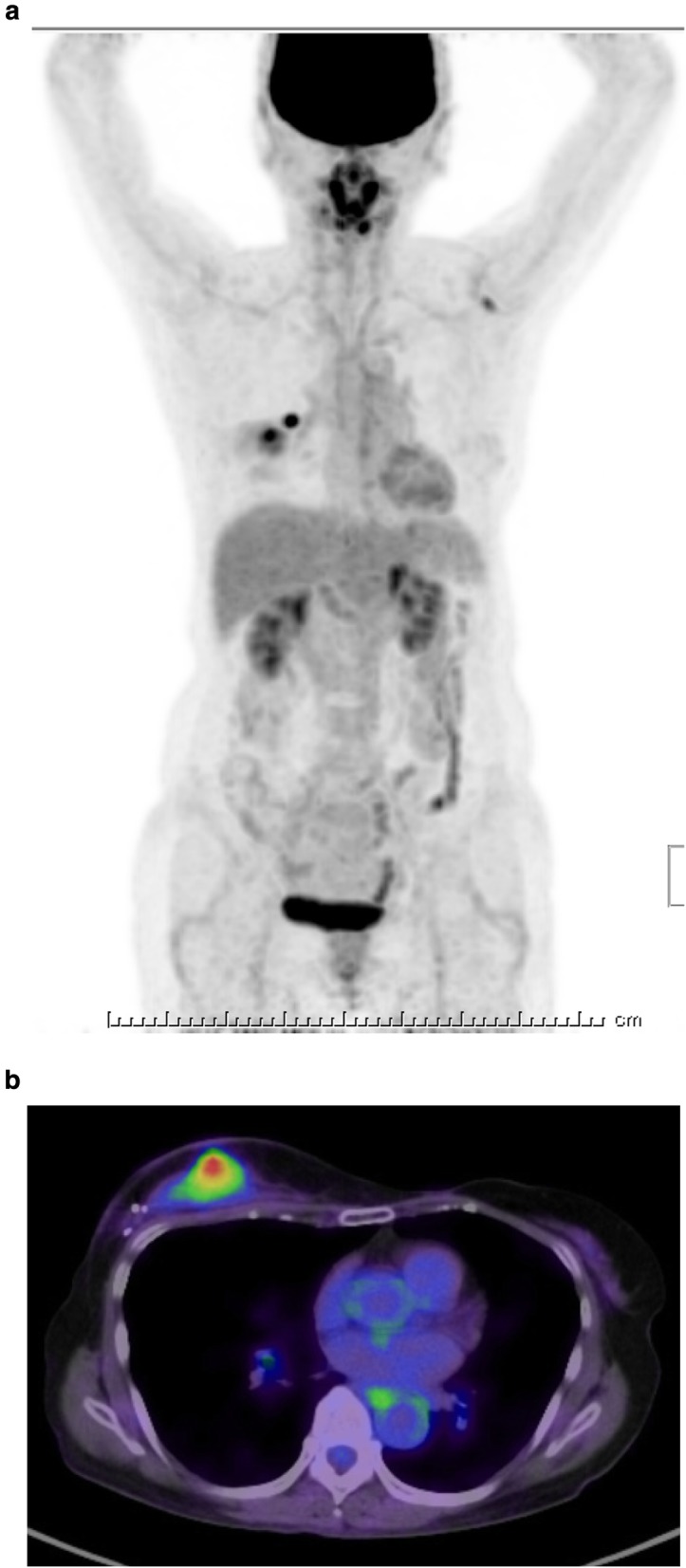
**a** PET/CT before the surgery showed no metastatic disease. **b** PET/CT showed accumulation in the tumor

**Fig. 4:**
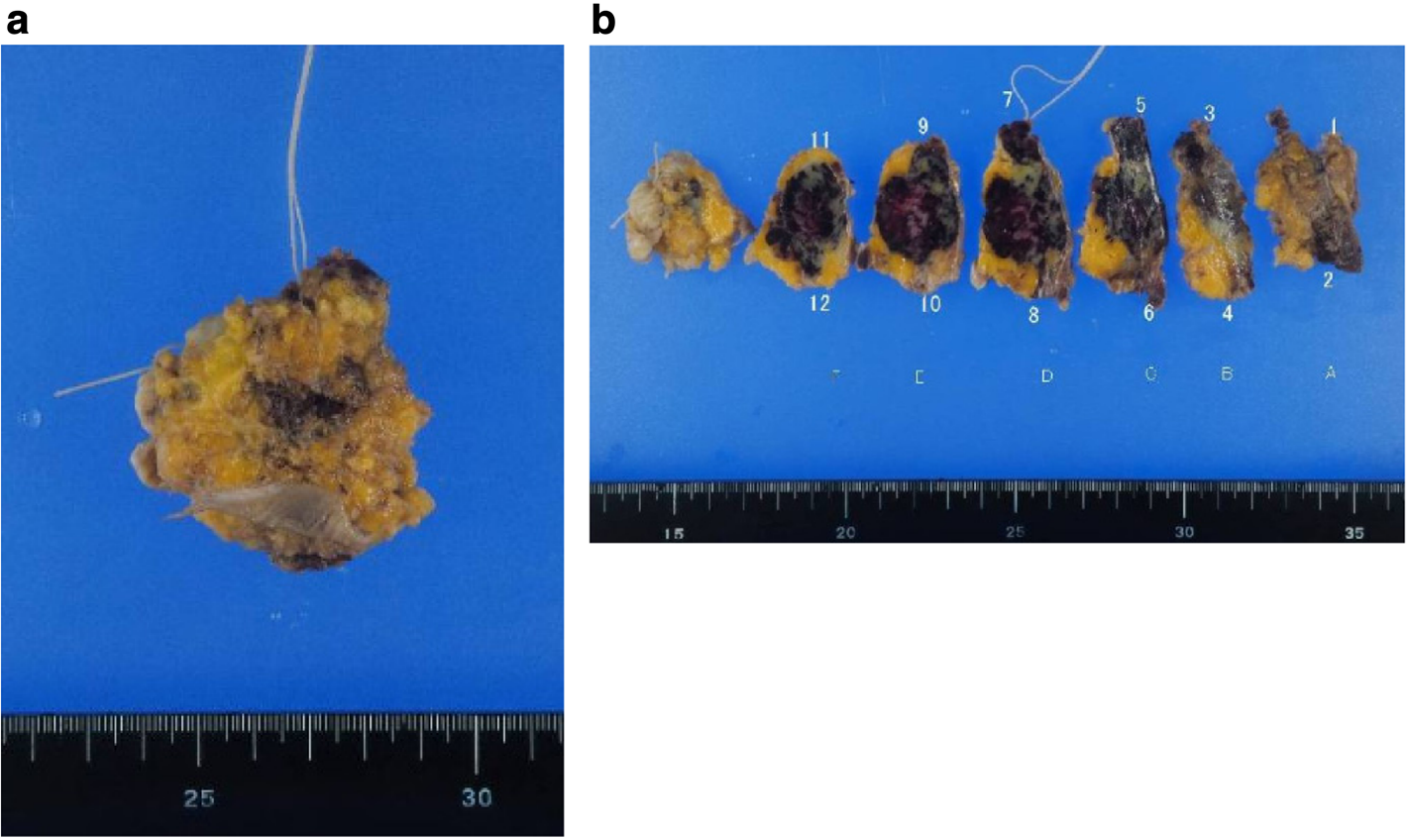
The excisional biopsy yielded the diagnosis of angiosarcoma. The resection margin was positive. **a** The whole sample. **b** The cross-section of the sample

**Fig. 5:**
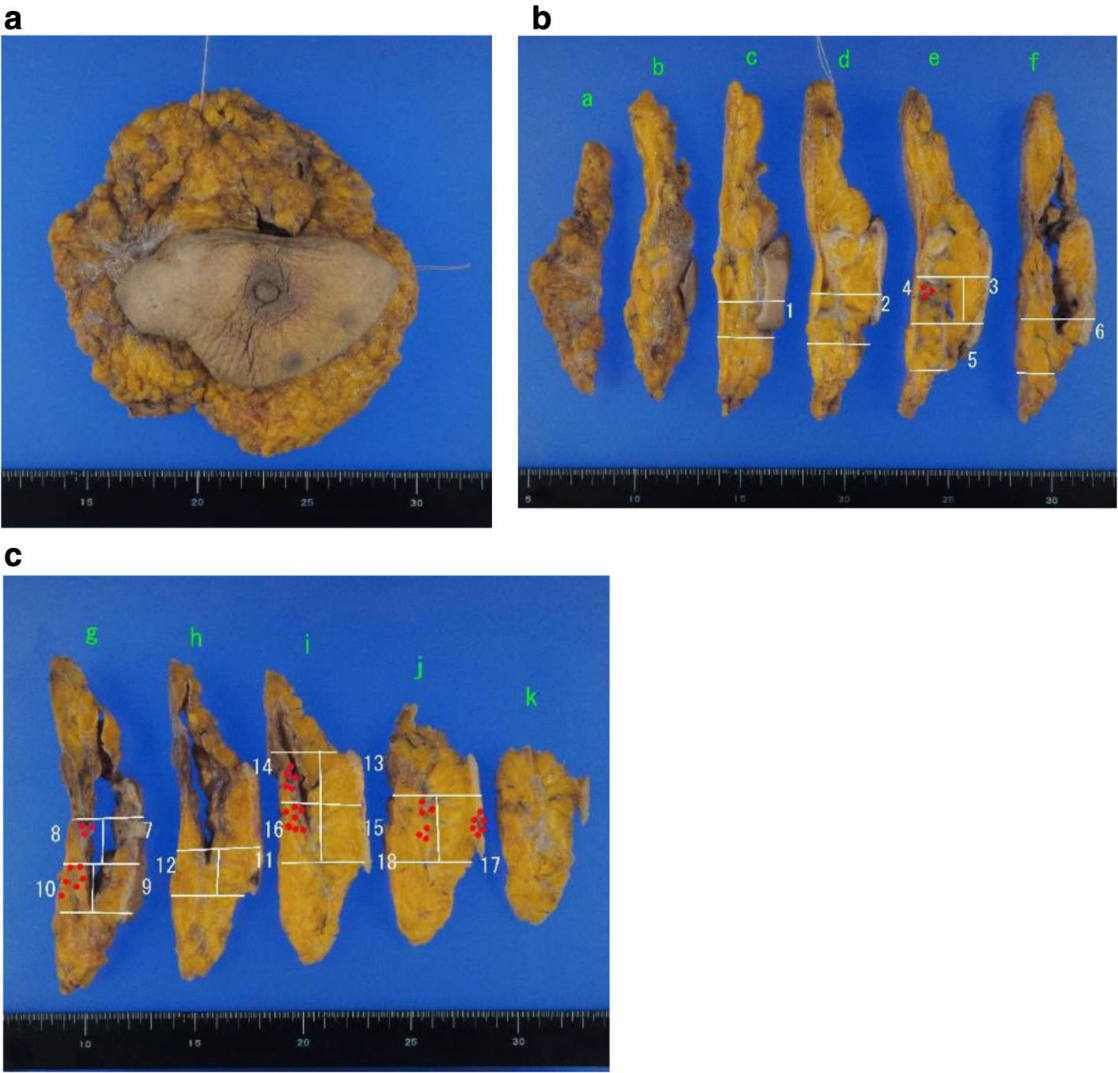
The patient underwent a right mastectomy. There was residual disease (red dotted area). The resection margin was negative. **a** The whole sample. **b**, **c** The cross-section of the sample

**Fig. 6:**
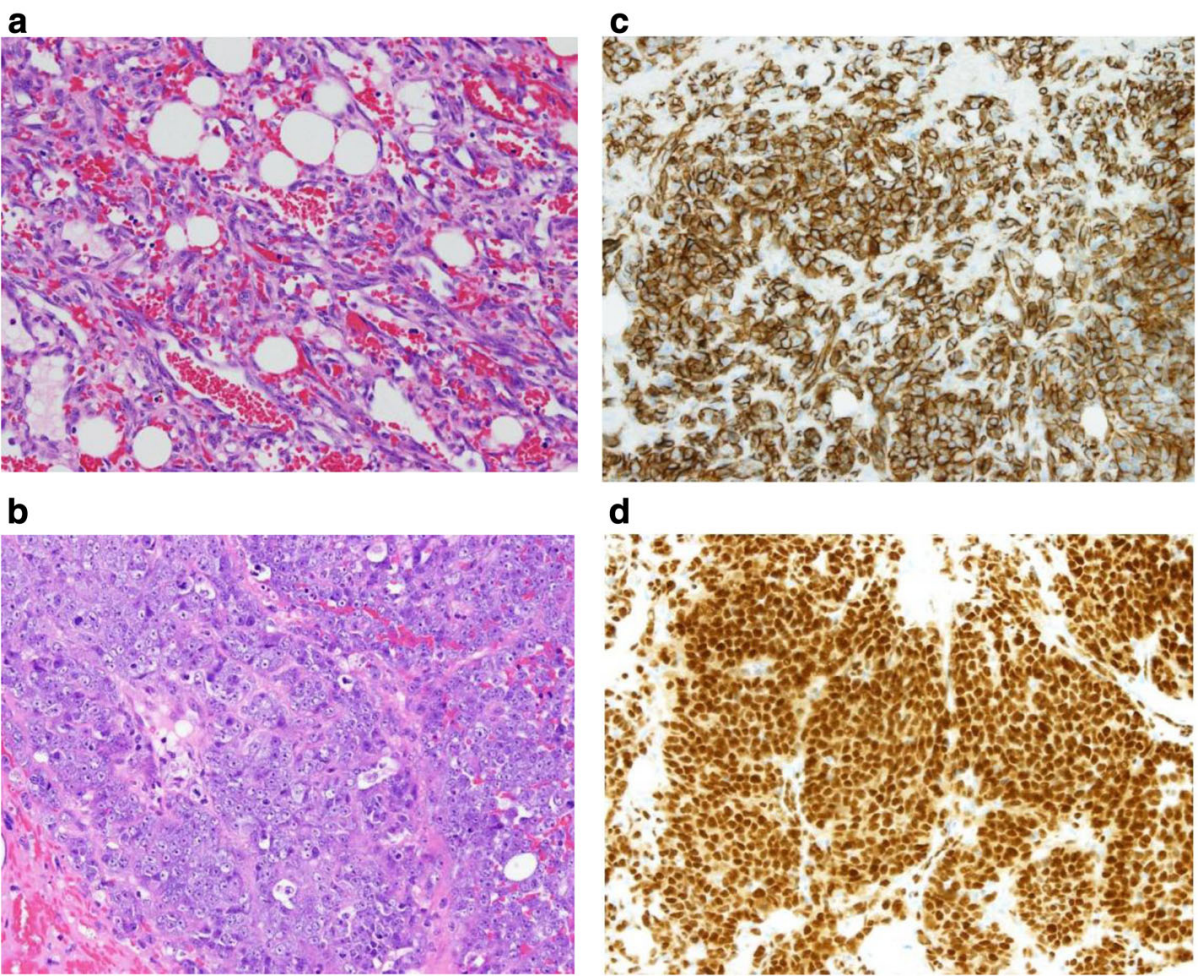
**a** Pathological analysis of the mastectomy revealed atypical cells that formed vascular spaces. (H-E) **b** Atypical cells proliferated. The cellularity of the block was very high. (H-E) **c** The endothelial cells showed strong positive staining with antibodies against CD31. **d** The nucleus showed strong positive staining with antibodies against EGFR

A 62-year-old woman had a history of right breast cancer and had undergone BCS. Pathologically, the tumor was classified as pT1cN0M0 pStageIA. Adjuvant RT (50 Gy) was administered to the residual breast tissue followed by hormone therapy (tamoxifen 20 mg daily for 5 years). There was no evidence of recurrence. However, 8 years after the first surgery, a tumor measuring 3 cm in size, appeared cutaneously in her right breast. The inside of the tumor had a low echoic area and the tumor borders were unclear on ultrasonography (Fig. 1a). CNB was performed twice and the bloody tissue was collected; however, it did not reveal a definitive diagnosis (Fig. 2a, b). Two months later, the tumor had grown bigger (Fig. 1b). CNB was performed again, but it did not reveal a definitive diagnosis. PET/CT showed accumulation in the tumor and no metastatic disease (Fig. 3a, b). We suspected that the tumor was angiosarcoma because of her medical history as well as the examination results. First, she had received RT after BCS. Second, the tumor had grown rapidly. Third, PET/CT had suggested the presence of malignant disease. She received an excisional biopsy and the pathological analysis yielded a diagnosis of angiosarcoma (Fig. 4). Subsequently, mastectomy was performed (Fig. 5) and the pathological analysis yielded a diagnosis of residual angiosarcoma (Fig. 6a, b) because of strong positive staining with antibodies against CD31 and EGFR (Fig. 6c, d). The tumor differentiation status was Federation Nationale des Centres de Lutte Contre le Cancer (FNCLCC) Grade 3 (tumor differentiation: score 3, mitotic count: score 3, tumor necrosis: score 0). The surgical margin was negative. We discussed the need for adjuvant chemotherapy using PTX. One month after the surgery, a nodule appeared on the skin around the surgical scar, which was confirmed as a recurrence of angiosarcoma by excisional biopsy. A few weeks later, a nodule reappeared on the skin around the surgical scar and was identified as a second recurrence of angiosarcoma by excisional biopsy. Subsequently, she underwent chemotherapy with PTX. There was no recurrence for 8 months following the second recurrence.

## Discussion

Angiosarcoma is an uncommon, malignant, blood vessel tumor. Angiosarcoma of the breast comprises 0.05% of all malignant tumors of the breast [2]. Cohen-Hallaleh et al. reported that the median age of diagnosis of the RIAS was 72 years (range 51–92 years) and the median time from completion of RT to the diagnosis of RIAS was 7.5 years (range 1–26 years) [1]. In our case, the age at diagnosis was lower than the median age, the duration from completion of radiation to the diagnosis of RIAS was similar to the median time. Patients with RIAS have a wide variety of cutaneous symptoms, for example, they experience purpura, erythema, ulcers, and edema. Because of the rarity of RIAS and the variety of symptoms, it is frequently misdiagnosed as a hematoma from injury, X-ray dermatitis [3], or contact dermatitis [4]. Cahan et al. established the criteria to diagnose RIAS which include the following: (1) history of RT, (2) asymptomatic latency period of several years (5 or more), (3) occurrence of sarcoma within a previously irradiated field, and (4) histologic confirmation of the sarcomatous nature [6]. The diagnosis from fine needle aspiration cytology (FNAC) or the CNB before radical surgery is very difficult. Strobbe et al. reported that the diagnosis rate of angiosarcoma by FNAC is 22% [7]. The reasons for this are as follows. First, angiosarcoma has a variety of morphological cellular features and in some cases, and it is misdiagnosed as a benign hemangioma [4]. Second, in contrast to primary angiosarcoma of the breast, malignant cells of recurring post-RT angiosarcoma typically have poorly differentiated nuclei with vesicular chromatin, prominent nucleoli, and mitotic activity [8]. Third, angiosarcoma of the breast is very rare. Therefore, if the definitive diagnosis could not be made from FNAC or CNB, excisional biopsy should be considered if criteria (1)–(3) are fulfilled. Our patient met the criteria (1)–(3) but did not fulfill criterion (4) before the excisional biopsy. However, we suspected angiosarcoma because the tumor had grown larger and PET/CT showed accumulation in the tumor. Regarding treatment as per the National Comprehensive Care Network (NCCN) guidelines, angiosarcoma is listed under soft tissue sarcoma [5]. The description of angiosarcoma itself is limited. The first choice of therapy is complete excision of the tumor with an adequate margin. Adjuvant RT should be considered for close soft tissue margins but it should not be routinely administered due to adverse events. In the NCCN guidelines for soft tissue sarcoma, adjuvant chemotherapy is not recommended for angiosarcoma. However, in patients with RIAS, some case reports have suggested that only surgical therapy is insufficient. There has been no study to investigate the effect of adjuvant chemotherapy on nonmetastatic, resectable angiosarcoma. There were a few case reports that discussed the effect of adjuvant chemotherapy on angiosarcoma. Nakamura et al. reported a case in which a woman, who was diagnosed with breast RIAS without metastasis, had undergone mastectomy and adjuvant chemotherapy of weekly PTX and the patient experienced no recurrence for 15 months [4]. On the contrary, Michigami et al. reported a case of a woman, who was diagnosed with primary angiosarcoma with no metastasis and had undergone conserving surgery, received IL-2 therapy, and experienced recurrence of angiosarcoma [9]. There is no prospective trial study to evaluate whether adjuvant chemotherapy should be recommended for resectable angiosarcoma. There is one prospective report about hyper-fractionated and accelerated RT. Tamara L. Smith et al. reported that hyper-fractionated and accelerated RT, with or without subsequent surgery, achieved higher rates of local control, disease-free survival, and overall survival of RIAS than the control [10]. Nicolas Panel et al. suggested that weekly PTX administration was well-tolerated and demonstrated clinical benefits in an ANGIOTAX study [11]. For metastatic soft tissue sarcoma, Winette T A van der Graaf et al. reported that Pazopanib prolonged progression-free survival as compared to the control [12]. In the NCCN guidelines, regimens such as docetaxel, PTX, and vinorelbine are recommended as systematic therapy for angiosarcoma [5]. In the NCCN guidelines for soft tissue sarcoma, resectable local recurrence should be excised and a decision regarding the use of radiation should be made case-by-case.

## Conclusion

Since it is difficult to diagnose angiosarcoma based on CNB or ABC, an excisional biopsy should be taken into consideration. In the case of angiosarcoma with no metastasis, adjuvant chemotherapy is not the usual mode of treatment unless there is a risk of recurrence. However, in patients who experience recurrence several times adjuvant chemotherapy may be considered to prevent distant metastasis.

## Data Availability

Not applicable.

## Abbreviations

BCS: Breast-conserving surgery
CNB: Core-needle biopsy
FNA: Fine needle aspiration
FNCLCC: Federation Nationale des Centres de Lutte Contre le Cancer
NCCN: National Comprehensive Cancer Network
PTX: Paclitaxel
RIAS: Radiation-induced angiosarcoma
RT: Radiotherapy
